# Computational tools for quantifying actin filament numbers, lengths, and bundling

**DOI:** 10.1242/bio.060267

**Published:** 2024-03-01

**Authors:** Laura A. Sherer, Biswaprakash Mahanta, Naomi Courtemanche

**Affiliations:** Department of Genetics, Cell Biology and Development, University of Minnesota, Minneapolis, MN 55455, USA

**Keywords:** Actin, Filament, Polymerization, Bundling, Kinetics, Fluorescence

## Abstract

The actin cytoskeleton is a dynamic filamentous network that assembles into specialized structures to enable cells to perform essential processes. Direct visualization of fluorescently-labeled cytoskeletal proteins has provided numerous insights into the dynamic processes that govern the assembly of actin-based structures. However, accurate analysis of these experiments is often complicated by the interdependent and kinetic natures of the reactions involved. It is often challenging to disentangle these processes to accurately track their evolution over time. Here, we describe two programs written in the MATLAB programming language that facilitate counting, length measurements, and quantification of bundling of actin filaments visualized in fluorescence micrographs. To demonstrate the usefulness of our programs, we describe their application to the analysis of two representative reactions: (1) a solution of pre-assembled filaments under equilibrium conditions, and (2) a reaction in which actin filaments are crosslinked together over time. We anticipate that these programs can be applied to extract equilibrium and kinetic information from a broad range of actin-based reactions, and that their usefulness can be expanded further to investigate the assembly of other biopolymers.

## INTRODUCTION

The actin cytoskeleton assembles into a diverse set of specialized structures that enable cells to carry out essential processes, including migration, growth, and division (Pollard and Cooper, 2009; Blanchoin et al., 2014). Individual actin filaments within these structures are crosslinked into distinct architectures, bound by specialized sets of actin-binding proteins, and ultimately disassembled in a regulated fashion (Blanchoin et al., 2014; Bartles, 2000). Given the complex interaction networks that govern the actin cytoskeleton, acquiring a comprehensive understanding of the mechanisms by which actin regulators cooperate to influence the assembly, functions and lifetimes of actin structures remains a challenge.

Much of what is known about actin dynamics has been learned through biochemical assays performed *in vitro* using purified proteins (Pollard, 2016). Notably, the use of light microscopy to visualize fluorescently labeled actin filaments and/or actin-binding proteins has provided detailed mechanistic insight at the single molecule, single filament, and filament network levels (Axelrod, 2001; Smith et al., 2014; Wioland et al., 2022; Ganzinger and Schwille, 2019). Quantification of time-series of fluorescence micrographs can yield rate constants and thermodynamic parameters that can be incorporated into molecular models (Ganzinger and Schwille, 2019; Pollard, 2016). However, the accuracy of these measurements is often complicated by the dynamic nature of the reactions. For example, the presence of overlapping filaments complicates the detection, resolution, and tracking of individual actin filaments in fluorescence micrographs. The number and lengths of polymerizing filaments also depend on rates of filament nucleation, elongation, annealing, and severing (Sept et al., 1999; Zweifel et al., 2021). It is therefore challenging to disentangle these processes to accurately track their evolution over time.

Here, we describe two programs written in the MATLAB programming language that facilitate counting, length measurements, and quantification of bundling of actin filaments visualized in fluorescence micrographs. Through automated detection, manual resolution of overlapping filaments, and adjustable background correction and fluorescence intensity thresholds, these programs enable accurate analysis of large numbers of filaments over the course of dynamic assembly reactions. Using an interactive interface, this set of programs enables users to tailor their analysis to a particular experiment. We have used these programs to quantify the length distributions of actin filaments assembled under a range of polymerization conditions and to obtain kinetic traces of bundling reactions in real-time (Sherer and Courtemanche, 2022; Zweifel et al., 2021). With minor modifications, we anticipate that these programs can be made applicable to the analysis of a broad range of polymer-based assays.

## RESULTS

We developed a set of MATLAB-based programs that enable quantification of the number, lengths, and bundling of fluorescently labeled actin filaments assembled *in vitro*. These programs can be used individually or in combination to generate equilibrium or kinetic measurements of actin filament nucleation, elongation and bundling. The inputs for these programs are fluorescence micrographs, which can be analyzed as individual frames or as a time-series stack of micrographs. We describe the programs using two representative reactions: (1) a sample containing pre-assembled actin filaments at equilibrium to demonstrate the determination of the total filament number and the measurement of filament lengths, and (2) a reaction in which actin filaments are cross-linked together over time to demonstrate the generation of kinetic measurements of bundling.

### Quantification of filament numbers and lengths

We recently showed that actin assembles into populations of filaments whose lengths at equilibrium depend on the polymerization conditions (Zweifel et al., 2021). To quantify filament lengths in a range of conditions, we generated a MATLAB-based program that can be used to detect and resolve individual actin filaments in fluorescence micrographs. We used this program to analyze populations of pre-assembled filaments that were generated by incubating 2 µM purified actin monomers in polymerization conditions at room temperature. At this concentration, monomers spontaneously nucleate into filaments, which elongate over time via continuous monomer binding at filament ends (Pollard, 1986). The reaction attained equilibrium following an incubation period of 2 h. We introduced fluorescein-isothiocyanate (FITC) phalloidin to stabilize and label the actin filaments, enabling their visualization by total internal reflection fluorescence (TIRF) microscopy.

Visualization of actin filaments by microscopy requires the immobilization of polymerization reactions on glass coverslip surfaces (Smith et al., 2014; Wioland et al., 2022). Filaments can be directly applied to the surface or introduced into a flow chamber. In the absence of buffer flow, actin filaments typically adhere to the surface in random orientations. This often results in filament crossover, which complicates the resolution and quantification of individual filaments. For this reason, we integrated automated error detection and user-based error correction into our program.

**Fig. 1.**
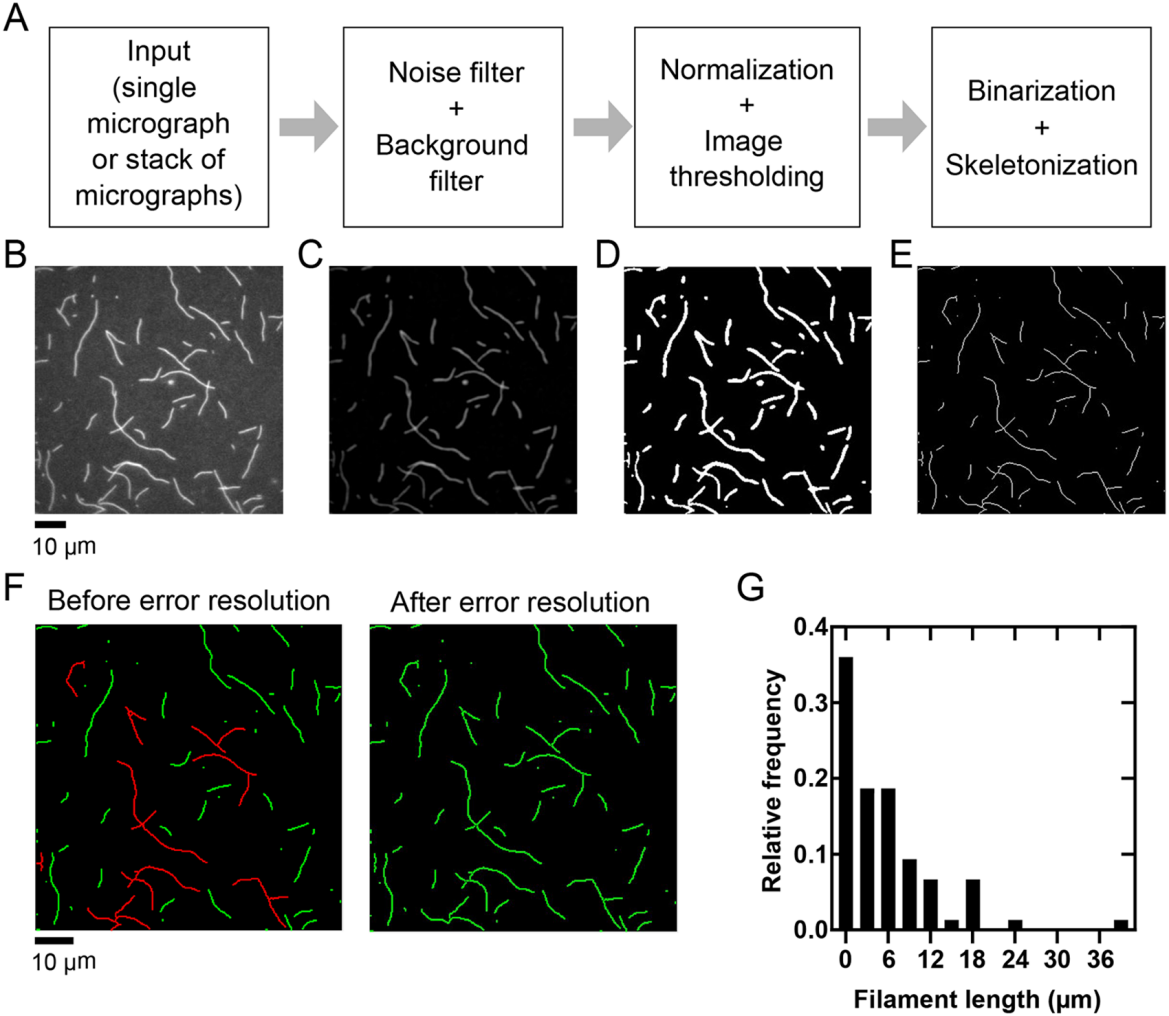
**Quantification of filament numbers and lengths.** (A) Flow-chart summarizing image processing and quantification steps. (B) Representative micrograph of filaments polymerized from 2 µM actin monomers, labeled with FITC-phalloidin, diluted into microscopy buffer, and imaged using TIRF microscopy. (C-E) Micrograph shown in B following sequential (C) noise filtering and background subtraction, (D) normalization and image thresholding, and (E) binarization and skeletonization. (F) (Left) Following image processing, overlapping filaments are detected as errors and highlighted in red. (Right) Each misidentified filament is processed individually until all errors are resolved, and all filaments are highlighted in green. (G) Histogram of filament lengths measured for the representative micrograph shown in panel B. The bin size is 3 µm.

Our analysis begins with the input of either a single micrograph or a time-series stack of micrographs (Fig. 1A,B). The selected image is first processed by noise filtering and background subtraction using two-dimensional Gaussian filters with standard deviations that are defined by the user (Fig. 1C). The image is normalized by setting the intensity of each pixel to a value between 0 and 1. To identify filamentous actin, pixels whose intensity values exceed a minimum threshold value set by the user are counted as ‘detected’, whereas all other pixels are undetected. A thresholding algorithm (implemented using MATLAB's ‘graythresh’ and ‘imbinarize’ functions (https://www.mathworks.com/help/images/ref/graythresh.html; https://www.mathworks.com/help/images/ref/imbinarize.html) is then applied to convert detected objects from a grayscale into binary images (Fig. 1D). This enables skeletonization, which reduces each two-dimensional filament into a line with a width of one pixel (Fig. 1E).

**Fig. 2.**
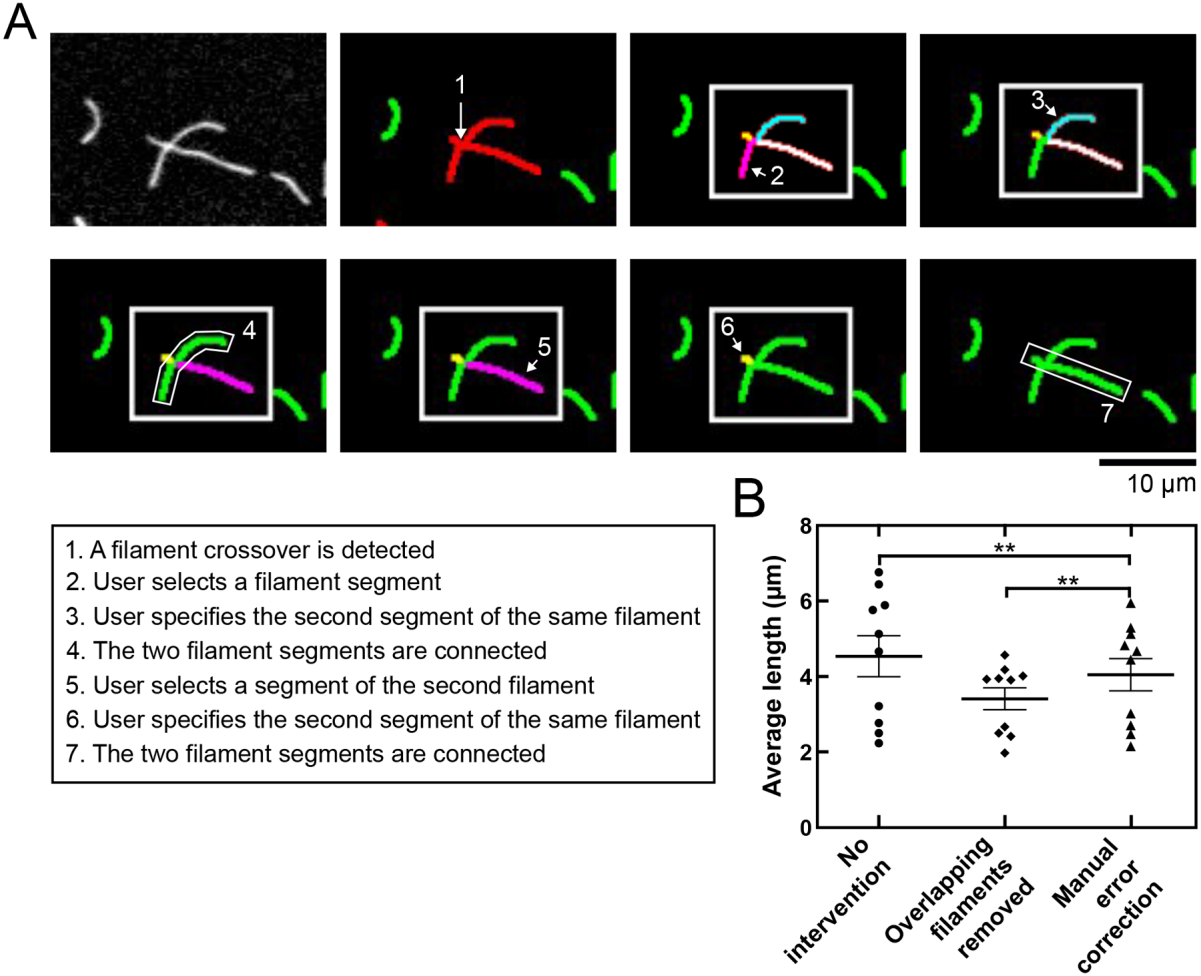
**Manual error processing enables resolution of overlapping filaments.** (A) Sequence of images depicting manual error correction. (Top left) Representative micrograph depicting two overlapping filaments. Step 1, the filaments are recognized by the program as an unresolved error. Steps 2-7, the user resolves the error by manually selecting segments comprising each individual filament. Each step in the process is summarized in the text box below the micrographs. (B) Average filament lengths quantified for ten micrographs collected at equilibrium following assembly of 2 µM actin monomers in identical polymerization conditions. Filament lengths were quantified in the absence of any error correction procedure (circles), with automated removal of overlapping filaments from the analysis (diamonds), and with manual error correction (triangles). ** indicates *P*<0.01 as determined using a paired *t*-test. Thick and thin lines indicate the mean and standard error for each data set.

Following skeletonization, each contiguous object is assessed, and its endpoints are quantified. Objects corresponding to individual filaments are detected as single lines, which possess two endpoints and lack a ‘branch point’. In contrast, objects that contain more than two endpoints or at least one branch point are identified by the program as containing detection ‘errors’ (Fig. 1F). Users may also manually select additional objects for correction. Filaments identified as containing detection errors are then resolved sequentially by the user through an interactive interface (Fig. 2A). Each segment (or ‘branch’) of the overlapping filaments is highlighted in a unique color, allowing the user to select a segment and specify how the error should be corrected (Fig. 2A, steps 2-7). The user is given the option to record the segment as a standalone filament or to combine the segment with another to form a single, continuous filament. This procedure can also be used to manually identify and remove instances of fluorescent noise that are erroneously detected as filamentous objects. Each misidentified filament is processed individually until all errors are resolved (Fig. 1F).

Following error detection and correction, filament lengths are quantified by dividing the perimeter of each object by two. Skeletonized filaments have a width of only one pixel, and thus the contribution of the filament's width to the perimeter is negligible. The measured lengths are converted from units of pixels to micrometers using a conversion factor determined by the properties of the camera and the magnification used to generate the supplied image. This conversion factor is specified by the user (see User Manual for further details). Length measurements can then be displayed in a histogram for direct observation and a table to enable export of data to facilitate further downstream analysis by the user (Fig. 1G).

Although our program is designed to be automated, the inclusion of a manual error correction step requires direct intervention from the user. To justify its inclusion, we compared average filament length measurements obtained with and without user-based error correction. In our analysis lacking user-based error correction, we assessed two alternative methods of processing misidentified objects: (1) automated removal of overlapping filaments from the analysis, and (2) the absence of any error correction procedure. We applied all three approaches to quantify filament lengths using the same set of ten micrographs, which were all obtained under the same experimental conditions (Fig. 2B). We found that automated removal of overlapping filaments produces shorter length measurements than those obtained using user-based error correction. This is consistent with an increase in the probability of filament overlaps with increasing filament length. In contrast, filament lengths measured in the absence of any error correction are consistently longer and show increased variability compared to those produced with user-based correction. Paired *t*-tests and Cohen's d analysis (which provides a standardized assessment of the magnitude of the difference between two data sets) indicate that the differences in the lengths obtained using each method are significant (*P*<0.01; d=0.33–0.82). Thus, user-based error correction significantly increases the accuracy of filament length measurements.

### Kinetic measurements of filament bundling

Actin filaments can be assembled into larger structures via the association of crosslinking or bundling proteins, which bind two filaments simultaneously (Michelot and Drubin, 2011). Bundling reactions are dynamic and progress at rates that depend on concentration and filament length (Sherer and Courtemanche, 2022; Falzone et al., 2012; Breitsprecher et al., 2011). When visualized *in vitro*, bundling has been shown to give rise to an increase in the fluorescence signal along the lengths of labeled actin filaments (Breitsprecher et al., 2011; Winkelman et al., 2016). As the reaction progresses, changes in the fluorescence intensity continue to occur along the lengths of the crosslinked filaments until equilibrium is reached (Sherer and Courtemanche, 2022). To quantify the progress of bundling reactions, we wrote a program that uses fluorescence intensity to detect single and crosslinked stretches of actin filaments. We used this program to generate kinetic bundling profiles for populations of fluorescently labeled, pre-assembled filaments following the introduction of fascin, an actin bundling protein. We collected a time series of fluorescence micrographs at 10 s intervals using TIRF microscopy.

**Fig. 3.**
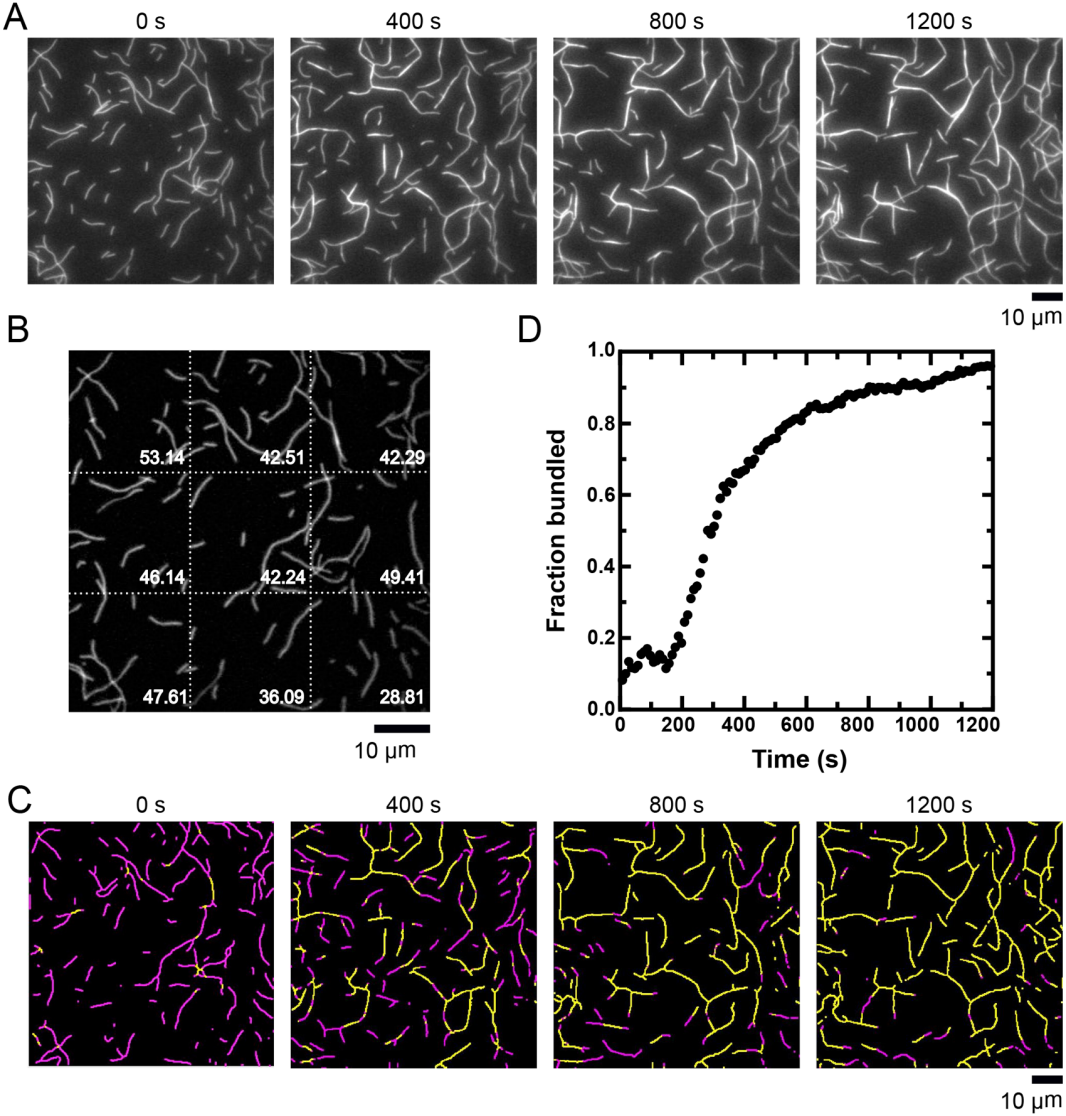
**Kinetic measurements of filament bundling.** Filaments were assembled from 2 µM actin monomers, labeled with FITC-phalloidin, and visualized by TIRF microscopy following the addition of 1.4 µM fascin. (A) Time series of micrographs depicting the progress of filament bundling. The same micrographs are also shown in Figure 4 to demonstrate the effects of image segmentation on the resolution of filaments and bundles. (B) Micrograph collected at time=0 s, before bundling has occurred. Following background subtraction, noise filtering, thresholding and skeletonization, the image has been segmented into a grid. The numbers correspond to the threshold fluorescence value (reported in arbitrary units of fluorescence intensity) above which pixels are considered bundled within each segment of the grid. (C) Automated detection of stretches of bundled (yellow) and single (magenta) filaments in each of the micrographs shown in panel A. (D) The fraction of the filamentous actin that is bundled over time for the reaction shown in panel A.

Analysis begins with the input of a time series of fluorescence micrographs in Audio Video Interleaved (AVI) format (Fig. 3A). The first frame is collected prior to the onset of bundling and therefore contains only individual filaments. This frame is used to set the initial threshold fluorescence intensity value above which pixels are considered bundled. To obtain these values, the micrograph is processed via noise filtering, background subtraction, thresholding, and skeletonization as described above.

Photobleaching over the course of a reaction decreases the fluorescence intensity of individual filaments. As a result, a threshold that enables resolution of individual and bundled filaments in the first frame of a reaction may not produce accurate bundle detection at later time points. To account for changes in the fluorescence signal that occur over time, a subset of micrographs in the reaction are subsequently analyzed. In each micrograph, the user is given the option to iteratively increase or decrease the threshold intensity to ensure that a representative sample of individual (i.e. not bundled) filaments is accurately detected across the whole image (see User Manual for further details). The threshold intensity values determined for each micrograph are then plotted. Linear and exponential fits are applied to extract a ‘photobleaching correction coefficient’, which is used to interpolate the appropriate threshold value for each micrograph in the reaction.

To account for uneven illumination in the micrograph, the image is segmented into a grid, and a threshold value is calculated for each rectangle (Fig. 3B). The threshold value is calculated using the following equation:
(1)

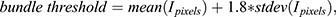
where I_pixels_ represents the intensities of all pixels corresponding to filaments in a given section of the noise-filtered, nonbinary image. Because the distribution of pixel intensities in each grid section is approximately normal, adding 1.8 standard deviations of the mean encompasses ∼93% of the pixel intensities corresponding to individual (or unbundled) filaments. The top ∼7% of the intensities are excluded from the threshold calculations because these values typically originate from pixels in which two or more overlapping filaments are present.

Following the determination of the bundle intensity threshold for each region of the first image, bundle assembly is quantified in all subsequent frames of the movie. Each image is processed as described above, and the intensity of each pixel is quantified. If a pixel's intensity falls below the specified threshold for the region in which it is found, it is registered as a single filament. If the intensity exceeds the threshold, it is registered as bundled. For each frame, the fraction of filamentous actin that is bundled is calculated by dividing the sum of the intensities of bundled pixels by the sum of all of the pixel intensities.

To enable the user to visually verify the accuracy of the analysis, the quantification results are synthesized into a movie (Fig. 3C). Each frame of the movie corresponds to a single micrograph. Pixels are highlighted in contrasting colors according to whether they are registered as a single filament or a bundle. Users should review the movie to visually assess the accuracy of bundle detection by verifying that a change in color occurs upon the coalescence of single filaments into bundles. If the analysis is deemed unsatisfactory, the threshold intensity value can be modified iteratively to improve the accuracy of bundle detection. To do so, we recommend adjusting the number of standard deviations that, when added to the mean pixel intensity, determines the basal fluorescence value above which filaments are registered as bundled (see Eqn 1 and the User Manual for additional details).

**Fig. 4.**
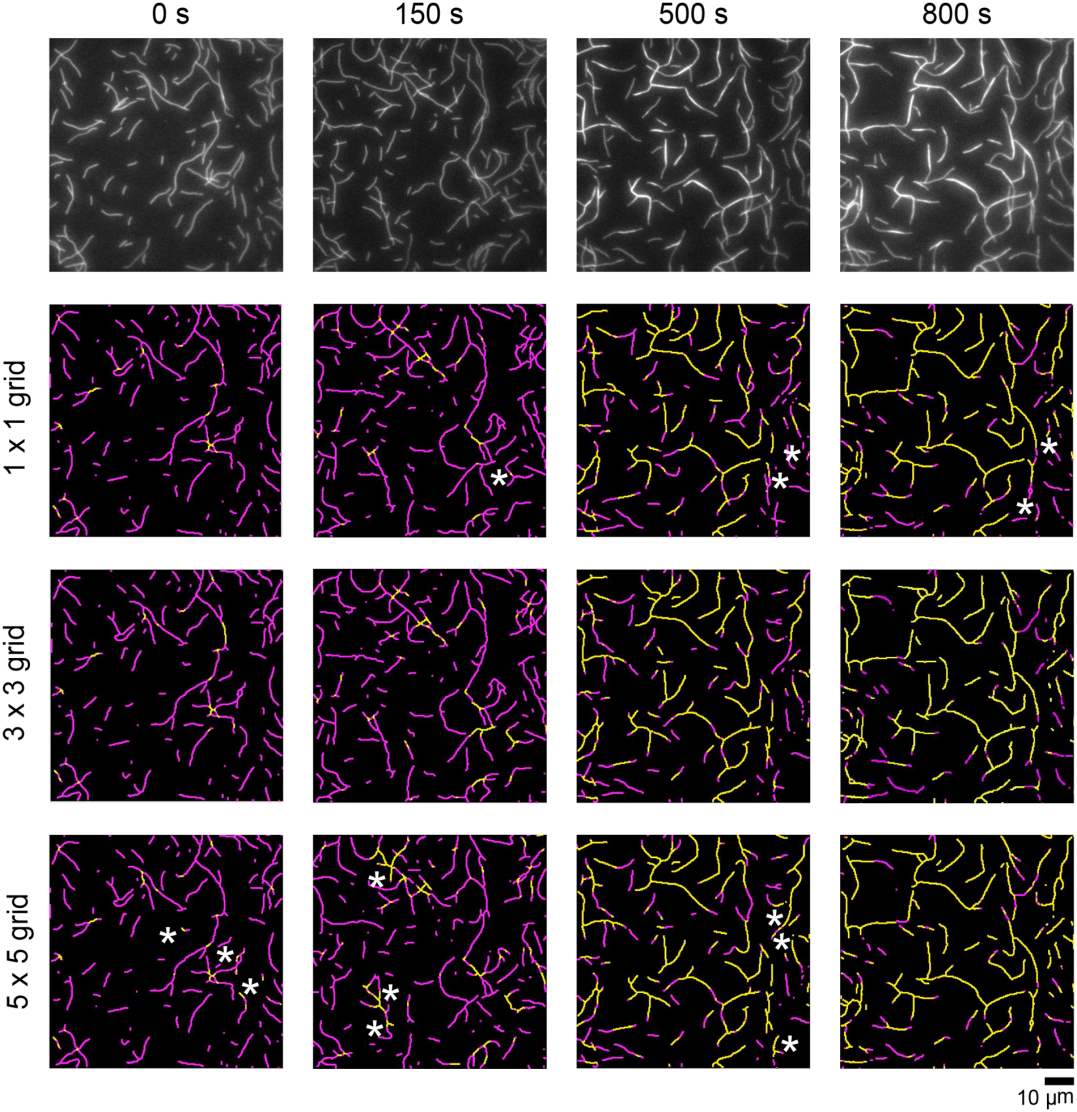
**Accurate resolution of filaments and bundles depends on micrograph segmentation.** Filaments were assembled from 2 µM actin monomers, labeled with FITC-phalloidin, and visualized by TIRF microscopy following the addition of 1.4 µM fascin. (Top row) Time series of micrographs depicting the progress of filament bundling. To enable direct comparison, the same micrographs are also shown in Figure 3. (Bottom rows) Automated detection of stretches of bundled (yellow) and single (magenta) filaments in each of the micrographs shown in the top row. The micrographs in each row were generated following segmentation into grids containing 1, 9 or 25 segments. Asterisks indicate representative instances where individual or bundled filaments are not accurately resolved.

The number of segments in the grid can also be adjusted by the user as needed to improve the accuracy of the analysis. To optimize the number of segments for our experiments, we compared analyzed micrographs generated using a range of grid sizes to raw micrographs collected over the course of a bundling reaction (Fig. 4). We found that increasing the number of grid segments improves filament detection in regions of the micrograph that are unevenly or dimly illuminated. On the other hand, the likelihood of erroneous bundle identification also increases with the number of grid segments. We determined that a 3×3 grid was optimal for our purposes.

The fraction of the actin that is incorporated into bundles is calculated for each micrograph according to the following equation:
(2)

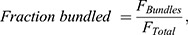
where F_Bundles_ is the sum of the intensities of the pixels whose fluorescence exceeds the threshold value, and F_Total_ is the sum of the intensities of all pixels in the micrograph. This metric accounts for ‘bundle expansion’ events in which additional filaments are incorporated into pre-existing bundles, as well as for variability in the amount of actin present in micrographs collected across different independent experiments (Sherer and Courtemanche, 2022). To enable extraction of kinetic parameters for the bundling reaction, the ‘Fraction bundled’ value can be plotted as a function of time using a time interval specified by the user (Fig. 3D).

## DISCUSSION

To facilitate the analysis of polymerization and bundling reactions, we generated two programs that enable the quantification of the number, lengths, and bundling status of actin filaments. We have validated the usefulness of our programs by applying them to detect and measure fluorescently labeled actin filaments both under equilibrium conditions and in a time-series of micrographs collected over the course of bundling reactions.

### Limitations and comparison with other analysis methods

Our programs require a filament fluorescence intensity that is high enough to set a threshold that captures the full length of the filament. This requirement can be easily met either by labeling filaments with fluorescent phalloidin or by labeling actin monomers with fluorescent dyes with high quantum yields. However, this requirement limits the applicability of our programs for the analysis of proteins that quench or otherwise modulate the fluorescence emission of actin filaments.

Filament length measurements are limited by the pixel size of the camera and the magnification used to collect the micrographs. Punctate objects smaller than one pixel in size may be detected by our programs, especially in experiments containing significant background noise (e.g. in experiments that contain fluorescently labeled actin monomers). However, owing to the inherent imprecision of measurements made on such small objects, the lengths of these detected puncta are not quantified by our programs. This reduces the contribution of spurious background noise to the analysis and improves the accuracy of the collective length measurements reported by the program. To further increase the accuracy of length measurements, we recommend that users adjust the concentration of filaments in their reactions to limit the incidence of multi-filament overlaps that may be challenging to resolve.

Quantification of the number of filaments that comprise bundles is also limited by the dynamic range of the camera that is used to collect the micrographs. When visualizing bundles that contain more than five filaments, we recommend directly labeling a fraction of the actin monomers with a fluorescent probe instead of using fluorescent phalloidin. Titration of the fraction of the monomers that are labeled will improve the accuracy of the measurements.

**Fig. 5.**
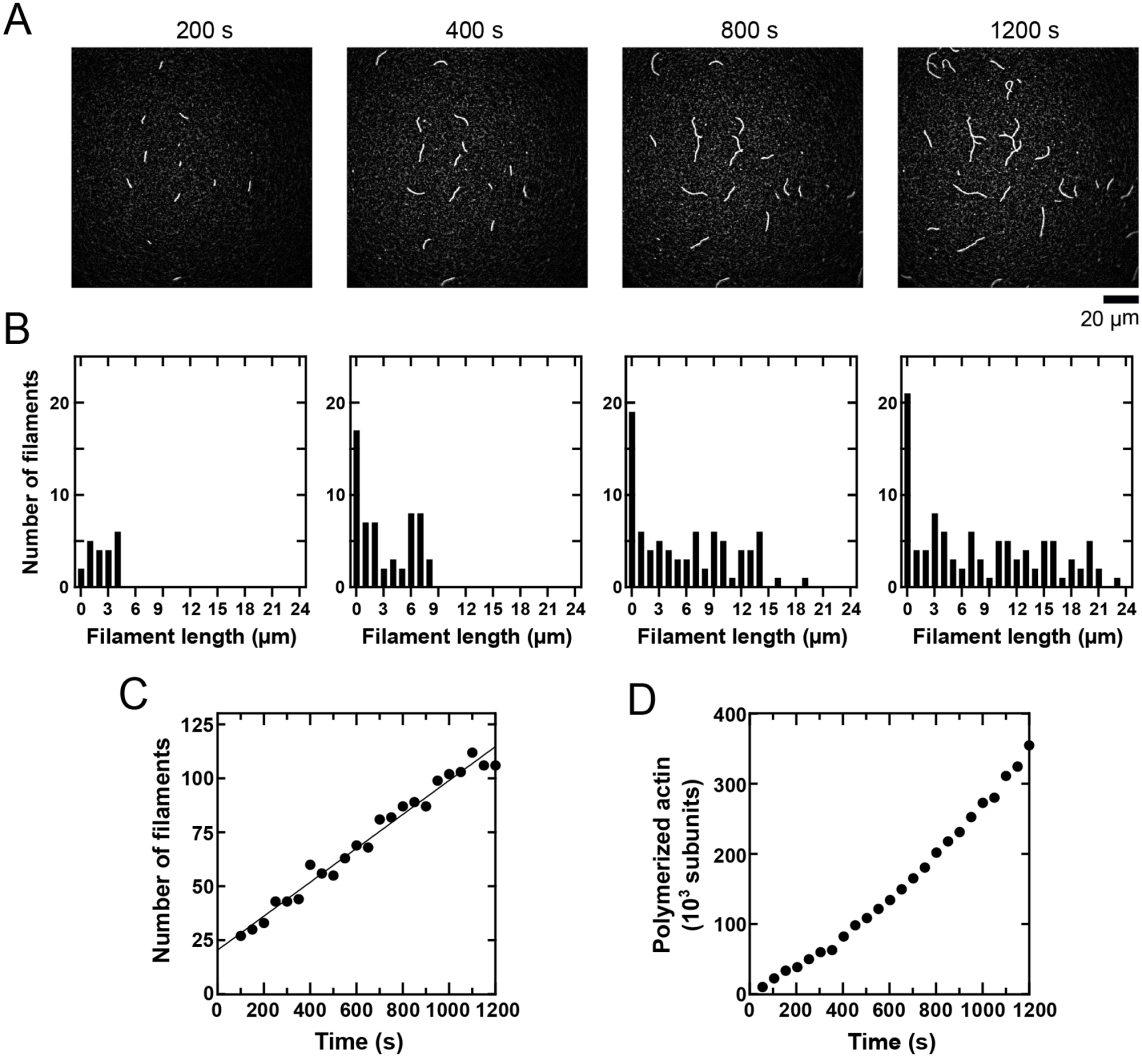
**Assessment of the overall progress of a polymerization reaction.** A solution containing 0.75 µM actin monomers (33% Oregon Green-labeled) in microscopy buffer was visualized by TIRF microscopy. (A) Time series of micrographs depicting the progress of an actin polymerization reaction. (B) Histograms of filament lengths corresponding to the micrographs shown in panel A. The bin size is 1 µm. (C) The number of filaments assembled over time in the polymerization reaction represented in panel A. Micrographs were collected and analyzed at 50-s time intervals. (D) The amount of polymerized actin that is contained in the reaction shown in panel A. Micrographs were collected and analyzed at 50-s time intervals.

Our programs do not allow tracking of individual filaments over time, nor do they provide direct measurements of the rates at which filament ends elongate. These measurements are best made either manually using ImageJ or with the use of plugins such as JFilament, which was developed to enable accurate tracking of filament ends over time (Smith et al., 2010). Despite this limitation, our programs can be used to assess the overall progress of a polymerization reaction (Fig. 5A). Assembly reactions are typically performed using fluorescently-labeled actin monomers, which give rise to a higher level of background signal than is observed in micrographs of filaments labeled with fluorescent phalloidin. The accuracy of filament detection depends on the signal-to-noise ratio of the image being analyzed, which is influenced by the experimental conditions and imaging settings. To account for higher background noise, we recommend iterative adjustment of the Gaussian filters that are applied during noise filtering and background subtraction to ensure accurate filament detection. This analysis will also require users to manually remove background artifacts that may be erroneously detected and identified as filamentous stretches by our programs. This can be done during the manual error correction process as described above. Users may also find it helpful to preprocess their micrographs using background subtraction algorithms that they employ in their standard image processing pipeline before subjecting them to analysis using our programs.

Following these adjustments, our program can be used to quantify the number of filaments that are assembled over time, as well as their length distribution (Fig. 5B,C). It should be noted that the number of filaments present in each micrograph does not necessarily correspond directly to the nucleation rate, but rather reports on the number of filaments that are long enough to become immobilized on the coverslip surface for visualization. With this limitation in mind, a time course of polymerization can be generated by integrating the distribution of filament lengths at each time point (Fig. 5D).

Despite the limitations outlined above, our programs possess several unique features that may be advantageous to users who seek to analyze populations of individual and bundled filaments. For example, automated detection enables rapid quantification of the lengths of large numbers of filaments. Similar measurements can be made manually or using ImageJ plugins which use pixel intensities and segmentation to determine the contours of individual filaments (Kuhn and Pollard, 2005; Smith et al., 2010). These methods are particularly well-suited for tracking the position of filament ends and quantifying changes in length over the course of polymerization. However, resolution of overlapping filaments using these methods can be laborious, as the detection of multiple bright pixels located in the vicinity of an overlap can generate inaccurate filament traces that must either be corrected or removed from the analysis. In our programs, the ability to manually resolve overlapping filaments enables precise identification of contiguous filament segments, thus simplifying the analysis process. Micrograph segmentation further facilitates bundle detection by quantifying local background noise to account for uneven illumination. This permits the simultaneous quantification of all individual and bundled filaments across an entire micrograph, rather than requiring the user to assess each filamentous object individually. Together, these features enable users to generate rapid and accurate measurements of ensembles of fluorescently labeled filaments assembled in a range of conditions.

### Further applications

We anticipate that our programs will have several applications beyond the uses described here. For example, these programs are not limited to the study of actin filaments and can be used to characterize the physical properties of other polymeric filament networks, including microtubules and intermediate filaments. They may also be used to analyze more complicated polymerization reactions performed in the presence of crosslinking proteins to quantify the time course of bundling as elongating filaments are incorporated into complex structures. Our programs can also be applied to quantify binding of fluorescently labeled proteins along the lengths of filaments and to study the effects of filament-binding proteins on filament and network dynamics. By enabling the analysis of complex polymeric reactions, we expect our programs to facilitate the expansion of current models of cytoskeletal dynamics.

## MATERIALS AND METHODS

### Protein purification

Actin was extracted from chicken muscle acetone powder and purified by one cycle of polymerization and depolymerization (Spudich and Watt, 1971). Monomers were separated from oligomers and filaments via gel filtration using Sephacryl S-300 resin (GE Healthcare) in G-buffer (2 mM Tris, pH 8.0, 0.2 mM ATP, 0.5 mM DTT, 0.1 mM CaCl_2_). For dynamic polymerization experiments, actin monomers were labeled with Oregon Green 488 maleimide (Thermo Fisher Scientific) prior to gel filtration. Human fascin-1 was expressed in BL21 (DE3) pLysS cells (Promega Corporation) from a pET21a plasmid that was modified to encode an N-terminal GST tag and a Tobacco Etch Virus (TEV) protease cleavage recognition sequence. Transformants were grown in 1 L of LB broth, induced at OD_600_ ∼0.6 with 0.5 mM isopropyl β-D-1-thiogalactopyranoside (IPTG), and shaken at 16°C overnight. Following cell harvest, the protein was purified as previously described (Sherer and Courtemanche, 2022).

### Actin polymerization and bundling

Ca^2+^-ATP-actin monomers were incubated with 0.05 mM MgCl_2_ and 0.2 mM EGTA for 3 min to generate Mg^2+^-ATP-actin. Polymerization was initiated by incubating 2 µM actin monomers in KMEI buffer [50 mM KCl, 1 mM MgCl_2_, 1 mM EGTA, 10 mM imidazole (pH 7.0)] for 2 h at room temperature. Assembled actin filaments were stabilized and fluorescently labeled via the addition of 4 µM fluorescein-isothiocyanate (FITC) phalloidin (Sigma-Aldrich). Following a 10-min incubation, samples were diluted to a final concentration of 10 nM actin in microscopy buffer {10 mM imidazole (pH 7.0), 50 mM KCl, 1 mM MgCl_2_, 1 mM EGTA, 50 mM DTT, 0.2 mM ATP, 15 mM glucose, 20 µg/mL catalase, 100 µg/mL glucose oxidase, 0.5% (w/v) methylcellulose [4000 cP at 2% (w/v)]}.

Samples were loaded onto imaging surfaces that were constructed by affixing Scotch tape (3M) around the perimeter of a 4.5 mm×4.5 mm area of glass coverslips (#1.5, 22 mm×50 mm) as described previously (Zweifel et al., 2021). Fascin was introduced to initiate bundling in a subset of reactions.

For dynamic polymerization reactions, flow chambers were assembled as previously described (Paul and Pollard, 2008; Sherer et al., 2018). The following solutions were introduced sequentially and allowed to incubate for 1 min: (1) 0.5% Tween 20 in HS-TBS [50 mM Tris (pH 7.5), 600 mM NaCl)], (2) ∼1 µM NEM-inactivated chicken skeletal muscle myosin (Kuhn and Pollard, 2005) in HS-TBS, and (3) 100 mg/mL BSA in HS-TBS. Chambers were washed with HS-TBS following each incubation step and with KMEI buffer prior to the introduction of a polymerization reaction. Mixtures of unlabeled and Oregon Green-labeled actin monomers were introduced into the flow chamber immediately following the addition of microscopy buffer to initiate polymerization.

In all experiments, filaments were visualized by through-objective TIRF microscopy on an Olympus Ti83 microscope equipped with a CellTIRF system using a 60x, 1.49 NA objective and a 488 nm laser. Images were acquired using a Hamamatsu C9100-23B ImagEM X2 EMCCD camera and CellSens Dimension software (Olympus America Inc.).

### Image analysis

The data analysis programs were created using MATLAB (The Mathworks Inc.) with the Image Acquisition, Image Processing, Signal Processing, and Curve Fitting Toolboxes enabled. Noise filtering and background subtraction were implemented by applying two-dimensional Gaussian blurs using MATLAB's ‘imfilter’ function. The width of each Gaussian blur was determined by multiplying the desired standard deviation by a factor of seven, which accounts for more than 99% of the pixels that may contribute to the signal in each region of the micrograph. Image thresholding, binarization and filament skeletonization were accomplished using MATLAB's ‘graythresh’, ‘imbinarize’, and ‘bwskel’ functions. Additional details can be found in our programs' user manual. Programs were compiled and tested using MATLAB versions 2020a, 2020b, and 2023a. MATLAB program files, sample image files, and a user manual are freely available through Github at the following website: https://github.com/biswa-mahanta/Actin-Filament-Bundling-Analysis.

## References

[BIO060267C1] Axelrod, D. (2001). Total internal reflection fluorescence microscopy in cell biology. *Traffic* 2, 764-774. 10.1034/j.1600-0854.2001.21104.x 11733042

[BIO060267C2] Bartles, J. R. (2000). Parallel actin bundles and their multiple actin-bundling proteins. *Curr. Opin. Cell Biol.* 12, 72-78. 10.1016/S0955-0674(99)00059-9 10679353 PMC2853926

[BIO060267C3] Blanchoin, L., Boujemaa-Paterski, R., Sykes, C. and Plastino, J. (2014). Actin dynamics, architecture, and mechanics in cell motility. *Physiol. Rev.* 94, 235-263. 10.1152/physrev.00018.2013 24382887

[BIO060267C4] Breitsprecher, D., Koestler, S. A., Chizhov, I., Nemethova, M., Mueller, J., Goode, B. L., Small, J. V., Rottner, K. and Faix, J. (2011). Cofilin cooperates with fascin to disassemble filopodial actin filaments. *J. Cell Sci.* 124, 3305-3318. 10.1242/jcs.086934 21940796 PMC4074248

[BIO060267C5] Falzone, T. T., Lenz, M., Kovar, D. R. and Gardel, M. L. (2012). Assembly kinetics determine the architecture of α-actinin crosslinked F-actin networks. *Nat. Commun.* 3, 861. 10.1038/ncomms1862 22643888 PMC3563296

[BIO060267C6] Ganzinger, K. A. and Schwille, P. (2019). More from less - bottom-up reconstitution of cell biology. *J. Cell Sci.* 132, jcs227488. 10.1242/jcs.227488 30718262

[BIO060267C7] Kuhn, J. R. and Pollard, T. D. (2005). Real-time measurements of actin filament polymerization by total internal reflection fluorescence microscopy. *Biophys. J.* 88, 1387-1402. 10.1529/biophysj.104.047399 15556992 PMC1305141

[BIO060267C8] Michelot, A. and Drubin, D. G. (2011). Building distinct actin filament networks in a common cytoplasm. *Curr. Biol.* 21, R560-R569. 10.1016/j.cub.2011.06.019 21783039 PMC3384529

[BIO060267C9] Paul, A. S. and Pollard, T. D. (2008). The role of the FH1 domain and profilin in formin-mediated actin-filament elongation and nucleation. *Curr. Biol.* 18, 9-19. 10.1016/j.cub.2007.11.062 18160294 PMC3712528

[BIO060267C10] Pollard, T. D. (1986). Rate constants for the reactions of ATP- and ADP-actin with the ends of actin filaments. *J. Cell Biol.* 103, 2747-2754. 10.1083/jcb.103.6.2747 3793756 PMC2114620

[BIO060267C11] Pollard, T. D. (2016). Actin and actin-binding proteins. *Cold Spring Harb. Perspect. Biol.* 8, a018226. 10.1101/cshperspect.a018226 26988969 PMC4968159

[BIO060267C12] Pollard, T. D. and Cooper, J. A. (2009). Actin, a central player in cell shape and movement. *Science* 326, 1208-1212. 10.1126/science.1175862 19965462 PMC3677050

[BIO060267C13] Sept, D., Xu, J., Pollard, T. D. and McCammon, J. A. (1999). Annealing accounts for the length of actin filaments formed by spontaneous polymerization. *Biophys. J.* 77, 2911-2919. 10.1016/S0006-3495(99)77124-9 10585915 PMC1300564

[BIO060267C14] Sherer, L. A. and Courtemanche, N. (2022). Cooperative bundling by fascin generates actin structures with architectures that depend on filament length. *Front. Cell Dev. Biol.* 10, 974047. 10.3389/fcell.2022.974047 36120572 PMC9479110

[BIO060267C15] Sherer, L. A., Zweifel, M. E. and Courtemanche, N. (2018). Dissection of two parallel pathways for formin-mediated actin filament elongation. *J. Biol. Chem.* 293, 17917-17928. 10.1074/jbc.RA118.004845 30266808 PMC6240877

[BIO060267C16] Smith, M. B., Li, H., Shen, T., Huang, X., Yusuf, E. and Vavylonis, D. (2010). Segmentation and tracking of cytoskeletal filaments using open active contours. *Cytoskeleton (Hoboken)* 67, 693-705. 10.1002/cm.20481 20814909 PMC3020657

[BIO060267C17] Smith, B. A., Gelles, J. and Goode, B. L. (2014). Single-molecule studies of actin assembly and disassembly factors. *Methods Enzymol.* 540, 95-117. 10.1016/B978-0-12-397924-7.00006-6 24630103 PMC4037564

[BIO060267C18] Spudich, J. A. and Watt, S. (1971). The regulation of rabbit skeletal muscle contraction. I. Biochemical studies of the interaction of the tropomyosin-troponin complex with actin and the proteolytic fragments of myosin. *J. Biol. Chem.* 246, 4866-4871. 10.1016/S0021-9258(18)62016-2 4254541

[BIO060267C19] Winkelman, J. D., Suarez, C., Hocky, G. M., Harker, A. J., Morganthaler, A. N., Christensen, J. R., Voth, G. A., Bartles, J. R. and Kovar, D. R. (2016). Fascin- and α-actinin-bundled networks contain intrinsic structural features that drive protein sorting. *Curr. Biol.* 26, 2697-2706. 10.1016/j.cub.2016.07.080 27666967 PMC5119644

[BIO060267C20] Wioland, H., Jégou, A. and Romet-Lemonne, G. (2022). Celebrating 20 years of live single-actin-filament studies with five golden rules. *Proc. Natl. Acad. Sci. USA* 119, e2109506119. 10.1073/pnas.2109506119 35042781 PMC8784122

[BIO060267C21] Zweifel, M. E., Sherer, L. A., Mahanta, B. and Courtemanche, N. (2021). Nucleation limits the lengths of actin filaments assembled by formin. *Biophys. J.* 120, 4442-4456. 10.1016/j.bpj.2021.09.003 34506773 PMC8553668

